# Advances in bacterial transcriptome understanding: From overlapping transcription to the excludon concept

**DOI:** 10.1111/mmi.14456

**Published:** 2020-03-17

**Authors:** Alejandro Toledo‐Arana, Iñigo Lasa

**Affiliations:** ^1^ Instituto de Agrobiotecnología, IdAB, CSIC‐Gobierno de Navarra Mutilva Spain; ^2^ Laboratory of Microbial Pathogenesis Navarrabiomed‐Complejo Hospitalario de Navarra (CHN)‐Universidad Pública de Navarra (UPNA), IdiSNA Pamplona Spain

**Keywords:** antisense RNA, excludon, noncontiguous operon, overlapping transcription, UTRs

## Abstract

In the last decade, the implementation of high‐throughput methods for RNA profiling has uncovered that a large part of the bacterial genome is transcribed well beyond the boundaries of known genes. Therefore, the transcriptional space of a gene very often invades the space of a neighbouring gene, creating large regions of overlapping transcription. The biological significance of these findings was initially regarded with scepticism. However, mounting evidence suggests that overlapping transcription between neighbouring genes conforms to regulatory purposes and provides new strategies for coordinating bacterial gene expression. In this *MicroReview*, considering the discoveries made in a pioneering transcriptome analysis performed on *Listeria monocytogenes* as a starting point, we discuss the progress in understanding the biological meaning of overlapping transcription that has given rise to the *excludon* concept. We also discuss new conditional transcriptional termination events that create antisense RNAs depending on the metabolite concentrations and new genomic arrangements, known as noncontiguous operons, which contain an interspersed gene that is transcribed in the opposite direction to the rest of the operon.

## Graphical abstract

Overlapping transcription between the mRNAs of the neighbouring genes is a common process in bacteria. The resulting paired RNAs are digested genome‐wide by the action of the double‐stranded endoribonuclease RNase III. New examples of gene arrangements where overlapping transcription plays a key role are summarised to explain our current understanding regarding how overlapping transcription may serve a general purpose of coordinating the bacterial gene expression.

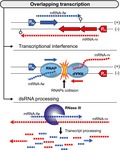

## 1 INTRODUCTION

It has been a decade since the publication of the first complete unbiased transcriptome analysis in the bacterial pathogen *Listeria monocytogenes* (Toledo‐Arana et al., 2009). This seminal study used high‐resolution tiling microarrays to investigate the transcriptional profiles of wild‐type and transcriptional regulatory mutants of *Listeria monocytogenes* grown in several conditions: (a) in vitro (exponential and stationary phase, hypoxia and low temperature); (b) ex vivo (human blood); and (c) in vivo (intestine of axenic mice). The results of the study anticipated a complex scenario in the bacterial genome transcription that has been confirmed by further studies in different bacteria (Cohen et al., 2016; Conway et al., 2014; Dornenburg, DeVita, Palumbo, & Wade, 2010; Kröger et al., 2012; Mitschke et al., 2011; Sharma et al., 2010; Thomason & Storz, 2010; Wade & Grainger, 2014). The bacterial transcriptome contains a substantial fraction of RNA sequences that overlap with other RNAs. For example, transcriptomes contain hundreds of noncoding regions, including *trans*‐acting small RNAs (sRNAs), *cis*‐acting antisense RNAs (asRNAs) and long 5′ and 3′ untranslated regions (5′ and 3′ UTRs) whose transcription start sites (TSSs) or transcription termination sites (TTSs) are often located inside the coding sequence of the neighbouring gene. This review will describe how the initial observations showing frequent overlapping between transcripts of the neighbouring genes in the *Listeria* transcriptome (Toledo‐Arana et al., 2009) have been enriched with new studies in other bacteria that are paving the way to the understanding of antisense transcription as a new mechanism to coordinate the bacterial gene expression.

### 1.1 Riboswitch‐dependent regulation of antisense RNAs

Riboswitches are regulatory elements that sense the fundamental metabolites or ions to control the expression of the genes encoding proteins involved in the metabolism or homoeostasis of these molecules (Winkler & Breaker, 2005). Despite the wide diversity of molecules that riboswitches are able to recognise, the regulatory activity of most of them is dedicated to modulating either transcription or translation by changing mutually exclusive RNA conformations. Regarding transcription attenuation, one of the alternative RNA structures serves as a Rho‐independent terminator while the other forms anti‐terminator hairpins that allow transcription. Analogously, translation could be inhibited or activated by alternative RNA structures that sequester or release ribosome‐binding sites (RBS), respectively. In both cases, the RNA structures are reorganised upon metabolite binding to select the appropriate one that will allow activation/inhibition of the required gene to respond according to the metabolite concentration (Serganov & Nudler, 2013).

**Figure 1:**
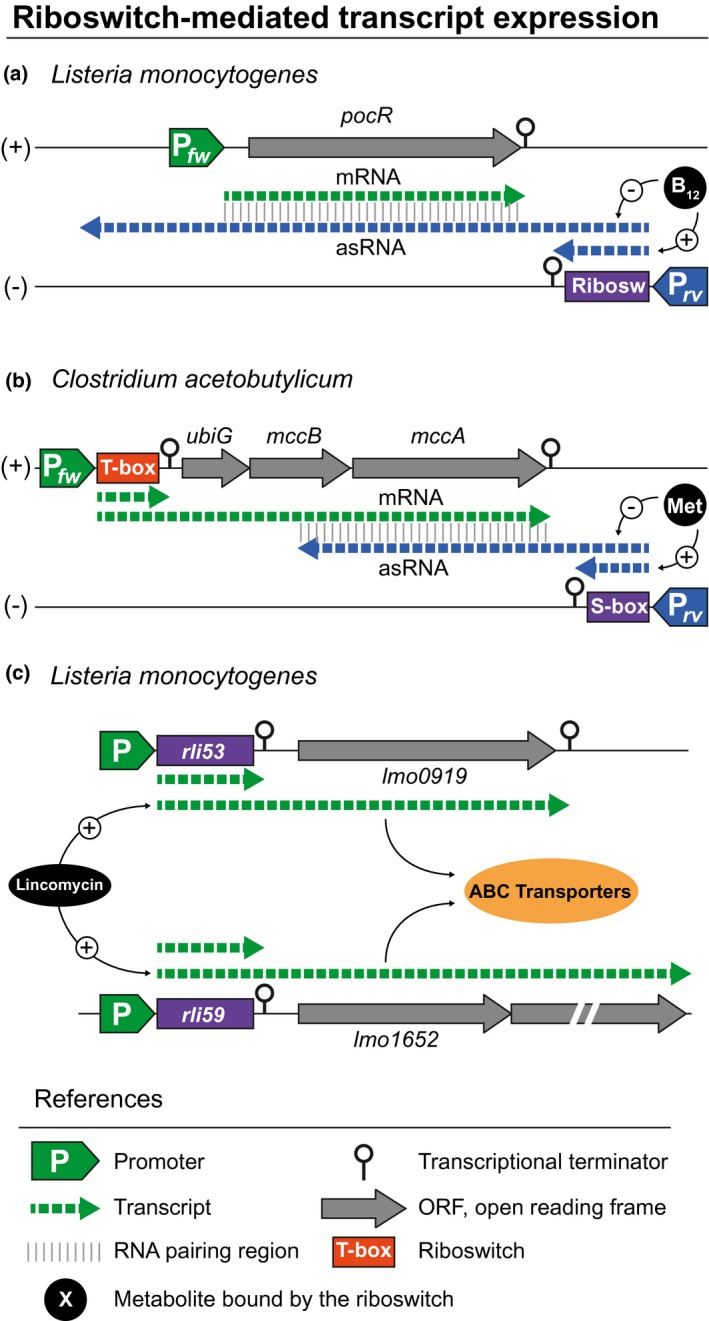
Examples of riboswitch‐dependent regulation. Schematic representation of genomic organisations, including riboswitches that control the transcription of antisense RNAs upon metabolite binding (a and b) or respond to the presence of antibiotics to activate the transcription of mRNAs encoding antibiotic resistance genes (c). Chart drawing references are included. (+) and (−) indicate forward and reverse DNA strands, respectively

The initial *L. monocytogenes* transcriptome analysis uncovered the transcription of a large number of long 5′ UTR containing riboswitches. As expected, most were located upstream of a coding sequence but, interestingly, some were transcribed in the opposite direction to a coding gene (Toledo‐Arana et al., 2009). One example of this configuration is *rli39*, a vitamin B_12_ riboswitch, that is positioned downstream of the *lmo1149* gene and in the opposite and convergent orientation to the next adjacent gene, *pocR* (*lmo1150*) (Toledo‐Arana et al., 2009). Depending on the vitamin B_12_ concentration, this riboswitch controls the transcription of an asRNA (*aspocR*) that overlaps *pocR* mRNA (Mellin et al., 2013) (Figure 1a). PocR is a transcriptional factor that, in the presence of propanediol, mediates propanediol catabolism by activating *pduCDE* genes. Propanediol catabolism requires a B_12_‐dependent diol dehydratase. Binding of vitamin B_12_ avoids *aspocR* transcription and, consequently, PocR protein is expressed promoting propanediol catabolism. In contrast, when there is not enough vitamin B_12_, the asRNA is expressed thereby inhibiting the PocR expression. Therefore, asRNA regulation by this riboswitch ensures that *pdu* genes are only expressed when the vitamin B_12_ cofactor required for propanediol catabolism is present (Mellin et al., 2013). A similar scenario where a riboswitch regulates the expression of an antisense transcript was previously described in *Clostridium acetobutylicum* (Andre et al., 2008) (Figure 1b). In this case, cysteine conversion from methionine is produced by the proteins encoded in the *ubiGmccBA* operon, whose expression is controlled by two functional convergent promoters associated with transcriptional antitermination systems, a cysteine‐specific T‐box and S‐box riboswitch, respectively. The S‐box riboswitch modulates the transcription of an asRNA overlapping the *ubiG* operon. Because the expression of this asRNA in *trans* did not affect the expression of the *ubiG* operon, the authors proposed a *cis*‐acting regulatory model via transcription interference at the *ubiG* locus (Andre et al., 2008).

Another important insight in the riboswitch field came from the application of Term‐seq methodology to *L. monocytogenes* (Dar et al., 2016). This method enables the quantitative mapping of all exposed RNA 3′ ends and allows the unbiased genome‐wide identification of genes that are regulated by premature transcription termination, including riboswitches (Dar et al., 2016). The application of Term‐seq to *L. monocytogenes* revealed that many of the previously annotated sRNAs were indeed *cis*‐acting regulatory 5′ UTRs. In particular, two sRNAs of unknown function, *rli53* and *rli59*, were found to function as antibiotic‐responsive riboregulators that control the expression of *lmo0919* and *lmo1652* genes, respectively, both encoding ABC transporter genes of unknown function (Figure 1c). Inspection of the regulatory 5′ UTR sequence of *lmo0919*, which is highly specific to lincomycin, revealed two alternative stem‐loop structures, a transcriptional terminator, and an antiterminator, respectively. Deletion of eight nucleotides from the antiterminator kept the regulator in a constitutively ‘closed’ state, even in the presence of lincomycin antibiotic, rendering the bacteria sensitive. In contrast, the deletion of eight nucleotides from the anti‐antiterminator released the antiterminator to interfere with the terminator structure. As a result, this mutation produced a constitutive read‐through (‘open’ state), even in the absence of antibiotics, increasing resistance to lincomycin. A three‐amino‐acid upstream open reading frame (uORF) exactly overlapping the inhibitory anti‐antiterminator sequence forms the basis for attenuation‐mediated regulation. The association of a ribosome with the antibiotic leads the ribosome to stall on the uORF, releasing the antiterminator to interfere with terminator folding and, thus, allowing read‐through into the antibiotic resistance gene (Dar et al., 2016). The application of Term‐seq in other model organisms (*Bacillus subtilis* and *Enterococcus faecalis*) and human oral microbiomes identified numerous riboswitches, suggesting that termination‐based regulation in response to antibiotics and other metabolites is very common in Gram‐positive bacteria (Dar et al., 2016). These considerations must be taken into account when studying sRNAs because, similar to what happens with *rli39*, *rli53* and *rli59*, it is likely that some of the annotated sRNAs are indeed riboswitches whose transcription terminates under specific environmental conditions in which the sRNA has been detected, while in a different condition, transcription continues generating a productive mRNA or asRNA.

### 1.2 Overlapping transcription between neighbouring genes

**Figure 2:**
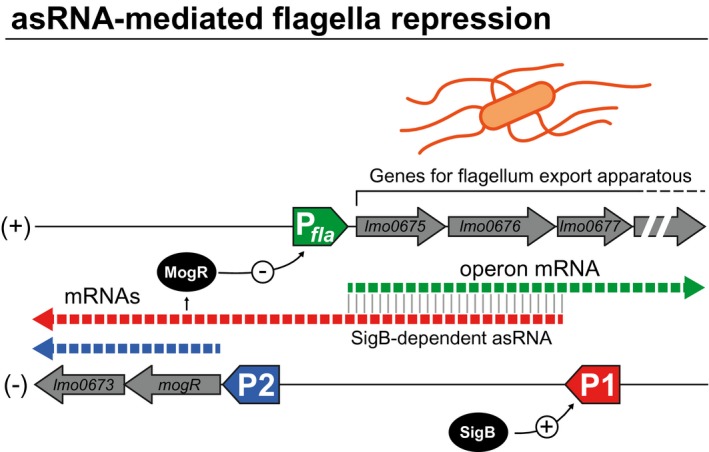
Paradigm of 5′ UTR‐overlapping‐mediated regulation. Schematic representation of the flagellum biosynthesis regulation in *L. monocytogenes*. P*fla* indicates the promoter region controlling the expression of the polycistronic mRNA transcript encoding the structural genes (*lmo0675* to *lmo0718*), which are required for building the flagellum export apparatus. P1 and P2 represent the promoter regions that drive the expression of the *mogR* and *lmo0673* genes. MogR is the transcriptional repressor of P*fla*. P1 is induced by the stress‐associated sigma factor SigB, and generates a long 5′ UTR that overlaps the flagellum operon. (+) and (−) indicates forward and reverse DNA strands, respectively

Another intriguing finding from the *L. monocytogenes* transcriptome was that often long 5′ or 3′ UTRs of well‐annotated genes overlap with neighbouring genes or UTRs (Toledo‐Arana et al., 2009). An example illustrating 5′ overlapping transcription corresponds to a long 5′ UTR of the *mogR‐lmo0673* operon (Figure 2). *L. monocytogenes* is highly flagellated and motile at low temperatures (30°C and below) but non‐motile at host‐related temperatures (37°C). Flagella biosynthesis requires dozens of genes included in a large operon (from *lmo0673* to *lmo0718*). Most of these genes are encoded in the positive DNA strand, with the exception of *lmo0673* and *mogR* (*lmo0674*), which are transcribed opposite to them. MogR is a transcriptional repressor that is essential for the temperature‐dependent transcription of motility genes (Gründling, Burrack, Bouwer, & Higgins, 2004). *Listeria* transcriptome data showed that the MogR protein is expressed from two alternative mRNAs that are transcribed from promoters P1 and P2 located 1,697 and 45 nucleotides upstream from the MogR start codon, respectively (Figure 2). Activation of the P1 promoter depends on the alternative stress‐associated sigma factor SigB, and generates a long 5′ UTR that overlaps the *lmo0675*, *lmo0676* and *lmo0677* genes that are required for flagella export apparatus (Figure 2). When the P1 transcript is overexpressed, the bacterial motility is reduced as a consequence of two complementary regulatory mechanisms. On the one hand, the asRNA pairing negatively regulates the expression of the flagella export apparatus genes. On the other hand, the P1‐derived transcript drives the expression of the MogR transcriptional repressor. Therefore, SigB‐activated long mRNA has a dual function by ensuring the repression of motility genes at both transcriptional and post‐transcriptional levels. Additional genomic configurations involving the overlapping of 5′ UTRs from genes with opposite or related functions have been described in several bacteria, indicating that the regulatory mechanism involving overlapping between the long 5′ UTRs of contiguous genes is widespread in bacteria (Cohen et al., 2016; Kopfmann, Roesch, & Hess, 2016; Lasa, Toledo‐Arana, & Gingeras, 2012; Wurtzel et al., 2012).

The next breakthrough in the field came from the transcriptome analysis of another Gram‐positive bacteria, *Staphylococcus aureus*, which demonstrated that overlapping RNA transcripts are digested genome‐wide by the action of the double‐stranded endoribonuclease RNase III (Lasa et al., 2011). High‐resolution transcriptome mapping was performed by combining the analysis of the total RNA fraction and the RNA fraction shorter than 50 nt. This methodological innovation turned out to be extremely useful for identifying the regions of the genome where overlapping transcription was taking place. The short RNAs displayed a symmetrical distribution in sense/antisense strands and accumulated in regions with noticeable overlapping transcription. This finding suggested that the collection of short RNAs was derived from the processing of the overlapping RNA transcripts by RNase III. This was confirmed by the analysis of the short RNA fraction of an RNase III mutant where the number of short RNA reads was drastically reduced. An important conclusion of this finding is that both sense and antisense overlapping transcripts have to be present simultaneously in the cytoplasm of the cell (Lasa et al., 2011; Lasa & Villanueva, 2014). Further, a study on *E. coli* indicated that overlapping sense/antisense transcripts are digested by RNase III (Lybecker, Zimmermann, Bilusic, Tukhtubaeva, & Schroeder, 2014). In this study, authors used a monoclonal antibody that recognises double‐stranded RNA (dsRNA) molecules to pull them down from a total RNA sample extracted from *E. coli* and its corresponding RNase III mutant. Sequencing of the purified dsRNAs showed that the transcripts of the dsRNA regions remain protected and more stable in the absence of an active RNase III. The majority of overlapping regions identified in this study (50%) correspond to the 5′ region of genes, whereas only 0.5% of the overlapping transcripts correspond to the 3′ region. Contrary to this, overlapping between 3′ UTRs of contiguous genes in *S. aureus* was found to be more frequent than overlapping between 5′ UTRs (Lasa et al., 2011; Ruiz de Los Mozos et al., 2013). Overlapping between 3′ UTRs occurred either because the transcriptional terminator of the gene was located far downstream of the end of the coding sequence or because the transcription continues by read‐through into the downstream gene. Read‐through transcription beyond the transcriptional terminator is more common in bacteria than previously anticipated and it can have important consequences in gene regulation. Application of a new RNA‐seq methodology (Yan, Boitano, Clark, & Ettwiller, 2018), named Smart‐Cappable‐seq, on *E. coli* has shown that 40% of transcription termination sites have read‐through that can alter the gene content of the define operons (http://biocomputo2.ibt.unam.mx/OperonPredictor/) (Taboada, Estrada, Ciria, & Merino, 2018). When the downstream genes were in the same direction, the extended transcript included at least an additional gene. This situation occurred in 34% of the known operons. In contrast, when the downstream genes were in the opposite direction, the extended transcript overlapped them, generating an antisense transcript.

The levels of pervasive read‐through transcription have been shown to be affected by the presence of the transcription terminator factor Rho (Bidnenko & Bidnenko, 2018). In *B. subtilis*, transcriptional and physiological studies demonstrated that the absence of Rho impairs the bacterial motility due to the extended transcription of genes that generate transcripts that overlap with neighbouring genes important for flagella apparatus, biofilm formation and sporulation (Bidnenko et al., 2017). Because the levels of Rho can fluctuate between cells and temporally, within a single cell, Rho‐dependent overlapping transcription can be a source of transcriptional noise in the bacterial population. In conclusion, the existence of overlapping transcription between 5′ and 3′ UTR of contiguous genes together with the existence of an RNase III‐dependent mechanism to process overlapping transcripts provides new evidence that the gene location in bacterial genomes obeys, in many cases, gene regulation criteria.

### 1.3 The excludon concept

**Figure 3:**
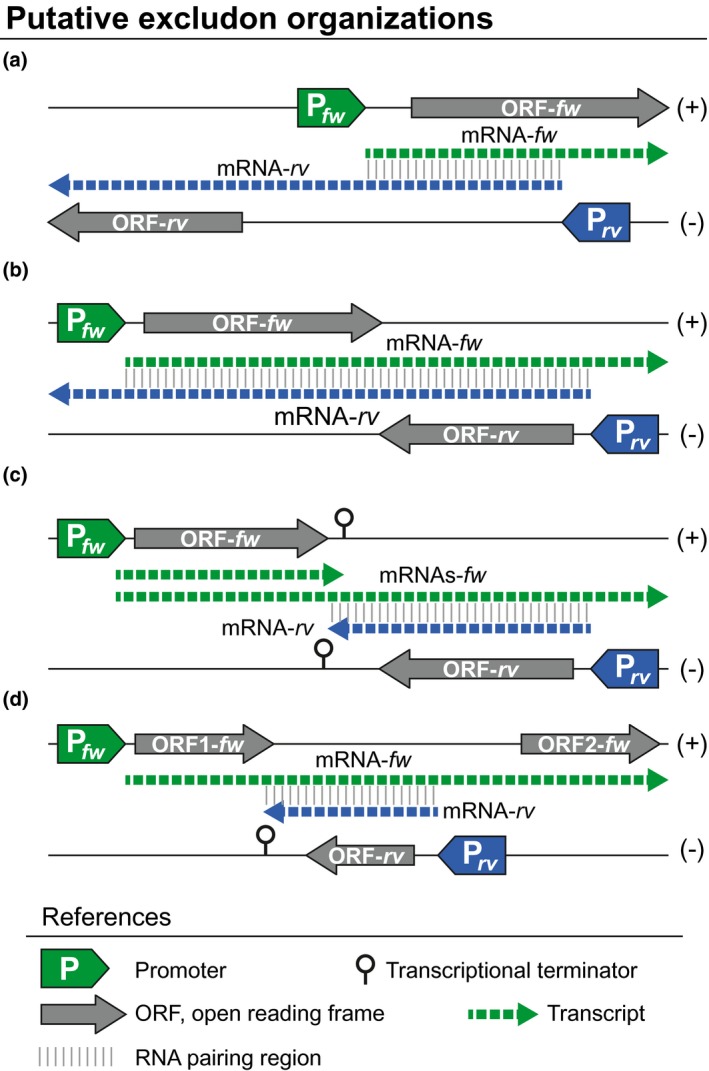
The excludon concept. Schematic representation of putative gene organisations that produce overlapping transcripts in bacteria. (a) Long 5′ UTR overlapping. (b) Long 3′ UTR overlapping among convergent genes that lack a transcriptional terminator between them. (c) Long 3′ UTR overlapping generated by transcriptional termination read‐through events. If a transcriptional terminator exists between two convergent genes, the RNA polymerase occasionally reads through the terminator signal generating long overlapping 3′ UTRs. (d) Noncontiguous operons that contain an interspersed gene that is transcribed in the opposite direction, generating two overlapping mRNAs that are reciprocally regulated. Chart drawing references are included. Different putative mRNA transcripts are represented as dashed arrows. (+) and (−) indicates DNA strands, respectively. P*_fw_* and P*_rv_* represent promoters encoded at the forward and reverse DNA strands, respectively

The finding that overlapping between 5′ and 3′ UTRs of contiguous genes is common in bacterial genomes together with the fact that RNase III digests sense/antisense overlapping transcripts inspired the Cossart's and Sorek's groups to propose a new paradigm of regulation based on overlapping transcription, termed ‘excludon’ (Sesto, Wurtzel, Archambaud, Sorek, & Cossart, 2013). The excludon concept describes the process by which the expression of a long mRNA transcript results in the repression of the expression of the overlapping transcript produced from the neighbouring gene. This genomic organisation allows the establishment of a regulatory relationship that results in the exclusive expression of the coding region that is expressed in higher amounts. The mRNAs of an excludon play a dual function as a coding mRNA and an asRNA. The most intuitive mechanisms for excludon‐mediated regulation is the processing of the dsRNAs by RNase III. However, other mechanisms, such as transcription interference, transcriptional attenuation or stabilisation of the RNAs after cleavage, are also possible. Examples of excludon transcriptional organisation have been described in different bacteria (Georg & Hess, 2018; Lasa et al., 2011; Lioliou et al., 2012; Quereda & Cossart, 2017; Sáenz‐Lahoya et al., 2019) (Figure 3). However, the exact mechanisms underlying the inhibitory effect of the overlapped transcripts in excludon organisation have just recently started being clarified.

**Figure 4:**
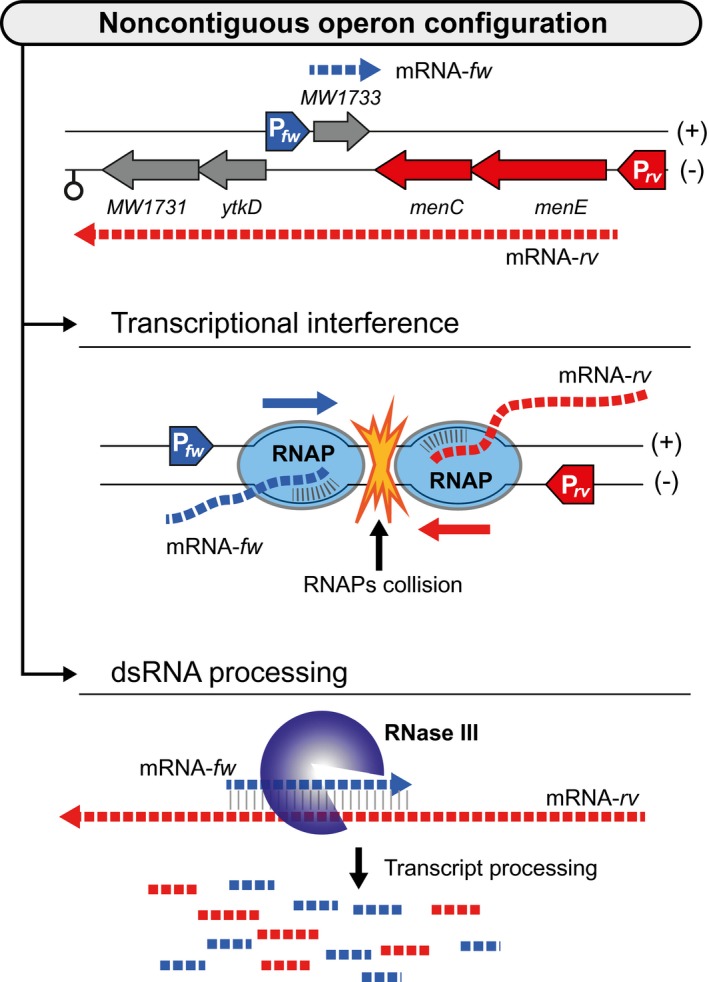
Example of a noncontiguous operon. Schematic representation of the noncontiguous *menE‐menC‐MW1733‐ytkD‐MW1731* operon architecture in *S. aureus*, which generates two overlapping mRNAs. The mRNA‐*fw* encodes the *MW1733* gene and the polycistronic mRNA‐*rv* includes the *menE‐menC‐ytkD‐MW1731* genes. The expression of these transcripts is reciprocally regulated by two complementary mechanisms: transcriptional interference by RNA polymerase (RNAP) collision and mRNA processing by double‐stranded endoribonuclease (RNase III). Overexpression of *MW1733* mRNA drives to a decreased MenE and MenC expression that results in the production of SCVs

An extreme example of gene regulation by overlapping transcription has been recently described in *S. aureus* (Sáenz‐Lahoya et al., 2019) (Figure 3d). The new transcriptional organisation, termed ‘noncontiguous operon’, consists of operons that contain a gene(s) that is transcribed in the opposite direction to the rest of the genes of the operon. The mRNA encoded on the opposite DNA strand to the operon serves as a bifunctional mRNA. It encodes for a protein while also acting as an asRNA, which base‐pairs all along its length with an internal untranslated region of the polycistronic mRNA. The noncontiguous operon architecture is exemplified by the genes *menE‐menC‐MW1733‐ytkD‐MW1731* involved in menaquinone synthesis in *S. aureus* (Figure 4). Transcriptome data indicated that *menE* and *menC* genes are co‐transcribed together with *yktD* and *MW1731* genes despite being separated by the *MW1733* gene, which is encoded in the opposite DNA strand (Figure 4). This operon configuration generates two mRNAs, the polycistronic *menE*‐*menC*‐*yktD*‐*MW1731* and the *MW1733* mRNA, which completely overlaps the polycistronic mRNA in the region between *menC* and *ytkD*. Therefore, the expression of both transcripts is reciprocally regulated by two complementary mechanisms, transcriptional interference and mRNA processing (Figure 4). The main evidence indicating the existence of transcriptional interference is that the expression of the tetracistronic operon was not affected when *MW1733* mRNA was expressed in *trans* from either a separate genomic location or a plasmid. Because pairing between complementary transcripts can occur regardless of whether they are expressed in *cis* or *trans*, the absence of regulation when both transcripts were produced in *trans* suggested that transcriptional interference plays a critical role. When transcription interference is overcome and both transcripts are produced, RNase III processes the mRNA duplex resulting in the cleavage of the tetracistronic mRNA into two independent transcripts with different half‐lives. On the one hand, the induction of *MW1733* mRNA expression leads to the reduction of MenE and MenC proteins that are included in the first half of the polycistronic transcript. On the other hand, RNA processing induces the stabilisation of the second half, including *ytkD* and *MW1731* genes. Reduction in the levels of MenE and MenC proteins resulted in a slowed growth phenotype characteristic of small colony variants (SCVs) (Sáenz‐Lahoya et al., 2019).

The synthesis of menaquinone, a component of the electron‐transport system (Bentley & Meganathan, 1982), illustrates well how the noncontiguous operon genetic arrangement may play a key role in the capacity of pathogenic bacteria to grow inside cells. Inhibition of the synthesis of menaquinone (or haemin) produces SCVs in *S. aureus* (Eiff et al., 1997). SCVs are usually isolated from patients experiencing chronic infections because bacteria showing this phenotype are able to persist better in mammalian cells and are less susceptible to aminoglycosides than their wild‐type counterparts (Proctor et al., 2014). The molecular mechanisms underlying the generation of SCVs remain, nonetheless, poorly understood because the subcultivation of SCVs in the laboratory reverts its phenotype to normal colony growth. The rapid switch between SCVs and normal cells strongly suggests that the phenotype is transient and is not mediated by genetic changes (Proctor et al., 2014).

**Figure 5:**
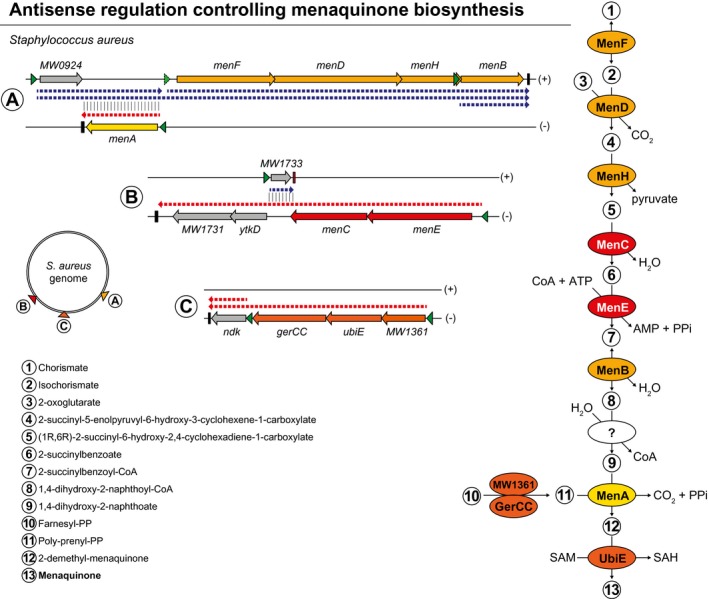
Genomic organisation of the genes required for menaquinone biosynthesis in *S. aureus*. The genes required for menaquinone biosynthesis are distributed in three operons (A, B and C) that are distantly encoded in the *S. aureus* genome (represented as a circle). A and B genomic regions are organised as noncontiguous operons. Triangles and rectangles represent putative promoters and transcriptional terminators, respectively. Different putative transcripts are shown as dashed arrows. (+) and (−) indicate forward and reverse DNA strands, respectively. The menaquinone biosynthetic pathway is represented at the right of the figure

The noncontiguous operon arrangement, including the *menE‐menC‐MW1733‐ytkD‐MW1731* genes, may provide a mechanism to shut down the synthesis of menaquinone and the generation of SCVs under environmental conditions where the expression of MW1733 is highly induced (Sáenz‐Lahoya et al., 2019) (Figure 4). The synthesis of menaquinone is accomplished by at least seven enzymes (MenA‐MenG) encoded in different operons (Hiratsuka et al., 2008). The *men* genes are organised in three operons that are distantly encoded in the *S. aureus* genome (Figure 5). Interestingly, a second noncontiguous operon configuration is found among *men* genes. Specifically, *MW0924* is co‐transcribed with *menFDHB* genes forming a long polycistronic transcript that overlaps the *menA* mRNA, which is encoded between *MW0924* and *menF* in the opposite direction (Sáenz‐Lahoya et al., 2019). It is tempting to speculate that both antisense transcripts will also be mutually regulated expanding the regulatory options to generate SCVs and normal‐growing bacteria depending on appropriate environmental signals.

Noncontiguous operon arrangements are not specific to *S. aureus*. Among the new enlarged operons identified by the group of L. Ettwiller in *E. coli*, there were some transcriptional arrangements consistent with the noncontiguous operon structure (Yan et al., 2018). Moreover, transcriptome analyses of different phages of *S. aureus* revealed that noncontiguous operon arrangements are also present in phage genomes (Chen et al., 2018; Quiles‐Puchalt et al., 2013). Together, these data indicate that noncontiguous organisation may be widespread in both Gram‐positive and Gram‐negative bacteria, as well as in bacteriophages.

### 1.4 Advantages of overlapping transcription‐mediated regulation

The antisense regulatory mechanisms derived from the initial observations in the *L. monocytogenes* transcriptome open the field of antisense gene regulation to the coordination of neighbouring gene expression in response to environmental cues. The consequences on the expression of a particular set of overlapping genes will depend on the differential activation of their promoters. In this regard, it is noteworthy that the stress‐associated sigma factor, SigB, regulates the transcription of one of the partners in several excludon configurations found in *L. monocytogenes* and *S. aureus* (Lasa et al., 2011; Toledo‐Arana et al., 2009; Wurtzel et al., 2012). In addition, several alternative factors, such as RpoN, RpoS, RpoH and SigX, are associated with the differential regulation of antisense transcripts in *Pseudomonas aeruginosa* (Eckweiler & Haussler, 2018). These findings reinforce the idea that specific physiological conditions activating one of the overlapping partners have consequences on the expression of the other partner. It would be interesting to determine what other transcriptional regulators are dedicated to control antisense expression. Considering that bacteria encodes hundreds of transcriptional regulators, it is likely that the expression of several asRNAs may be induced in specific conditions by transcriptional regulators.

It is also important to highlight that few genomic changes are sufficient to create novel antisense regions. For example, few nucleotide mutations can create or modify promoters and transcriptional terminator signals. Another advantage of the regulation mediated by overlapping transcription is that it permits the evolution of each of the overlapping genes by nucleotide changes that simultaneously affect both mRNA transcripts without altering their binding affinity (Brantl, 2015). From an evolutionary perspective, this has important consequences because it allows changes in the genome that affect, for instance, the promoter region of one of the partners while preserving the regulatory mechanisms. Unsurprisingly, bacteria have taken advantage of such versatility and make widespread use of overlapping transcription to coordinate gene expression.

## 2 FINAL REMARKS

Breakthroughs in methods to analyse total bacterial RNA content (tiling array and RNA‐seq sequencing technologies) lead to the complete characterisation of transcriptomes with a precision previously unimaginable. A limitation of these methods, due to the requirement of a minimal amount of RNA for the analysis, is that they are conducted at a ‘population level’, with the resulting transcriptome being an average of the transcriptomes of millions of prokaryotic cells (Kang, McMillan, Norris, & Hoang, 2015; Saliba, Santos, & Vogel, 2017; Saliba, Westermann, Gorski, & Vogel, 2014). Therefore, specific patterns of gene expression that occur in one cell (correlation of the expression of the sense/antisense mRNAs in noncontiguous operons) are diluted among cell‐to‐cell heterogeneity within the whole population. We foresee that the next technological breakthrough to progress the knowledge in antisense‐mediated regulation will be related to the capacity to analyse bacterial transcriptomes at a single cell level.

## CONFLICT OF INTEREST

The authors declare to have no conflict of interest.

## References

[mmi14456-bib-0001] André, G. , Even, S. , Putzer, H. , Burguière, P. , Croux, C. , Danchin, A. , … Soutourina, O. (2008). S‐box and T‐box riboswitches and antisense RNA control a sulfur metabolic operon of *Clostridium acetobutylicum* . Nucleic Acids Research, 36, 5955–5969. 10.1093/nar/gkn601 18812398 PMC2566862

[mmi14456-bib-0002] Bentley, R. , & Meganathan, R. (1982). Biosynthesis of vitamin K (menaquinone) in bacteria. Microbiological Reviews, 46, 241–280. 10.1128/MMBR.46.3.241-280.1982 6127606 PMC281544

[mmi14456-bib-0003] Bidnenko, E. , & Bidnenko, V. (2018). Transcription termination factor Rho and microbial phenotypic heterogeneity. Current Genetics, 64, 541–546. 10.1007/s00294-017-0775-7 29094196

[mmi14456-bib-0004] Bidnenko, V. , Nicolas, P. , Grylak‐Mielnicka, A. , Delumeau, O. , Auger, S. , Aucouturier, A. , … Bidnenko, E. (2017). Termination factor Rho: From the control of pervasive transcription to cell fate determination in *Bacillus subtilis* . PLoS Genetics, 13, e1006909 10.1371/journal.pgen.1006909 28723971 PMC5540618

[mmi14456-bib-0005] Brantl, S. (2015). Antisense‐RNA mediated control of plasmid replication—pIP501 revisited. Plasmid, 78, 4–16. 10.1016/j.plasmid.2014.07.004 25108234

[mmi14456-bib-0006] Chen, J. , Quiles‐Puchalt, N. , Chiang, Y. N. , Bacigalupe, R. , Fillol‐Salom, A. , Chee, M. S. J. , … Penadés, J. R. (2018). Genome hypermobility by lateral transduction. Science, 362, 207–212. 10.1126/science.aat5867 30309949

[mmi14456-bib-0007] Cohen, O. , Doron, S. , Wurtzel, O. , Dar, D. , Edelheit, S. , Karunker, I. , … Sorek, R. (2016). Comparative transcriptomics across the prokaryotic tree of life. Nucleic Acids Research, 44, W46–W53. 10.1093/nar/gkw394 27154273 PMC4987935

[mmi14456-bib-0008] Conway, T. , Creecy, J. P. , Maddox, S. M. , Grissom, J. E. , Conkle, T. L. , Shadid, T. M. , … Wanner, B. L. (2014). Unprecedented high‐resolution view of bacterial operon architecture revealed by RNA sequencing. mBio, 5, e01442‐14 10.1128/mBio.01442-14 25006232 PMC4161252

[mmi14456-bib-0009] Dar, D. , Shamir, M. , Mellin, J. R. , Koutero, M. , Stern‐Ginossar, N. , Cossart, P. , & Sorek, R. (2016). Term‐seq reveals abundant ribo‐regulation of antibiotics resistance in bacteria. Science, 352, aad9822 10.1126/science.aad9822 27120414 PMC5756622

[mmi14456-bib-0010] Dornenburg, J. E. , DeVita, A. M. , Palumbo, M. J. , & Wade, J. T. (2010). Widespread antisense transcription in *Escherichia coli* . mBio, 1, e00024‐10 10.1128/mBio.00024-10 20689751 PMC2912661

[mmi14456-bib-0011] Eckweiler, D. , & Haussler, S. (2018). Antisense transcription in *Pseudomonas aeruginosa* . Microbiology, 164, 889–895. 10.1099/mic.0.000664 29738307 PMC6097033

[mmi14456-bib-0012] Georg, J. , & Hess, W. R. (2018). Widespread antisense transcription in prokaryotes. Microbiology Spectrum, 6(4), 1–20. https://doi.org/10.1128/microbiolspec.RWR‐0029‐2018 10.1128/microbiolspec.rwr-0029-2018 PMC11633618 30003872

[mmi14456-bib-0013] Gründling, A. , Burrack, L. S. , Bouwer, H. G. A. , & Higgins, D. E. (2004). *Listeria monocytogenes* regulates flagellar motility gene expression through MogR, a transcriptional repressor required for virulence. Proceedings of the National Academy of Sciences of the United States of America, 101, 12318–12323. 10.1073/pnas.0404924101 15302931 PMC514476

[mmi14456-bib-0014] Hiratsuka, T. , Furihata, K. , Ishikawa, J. , Yamashita, H. , Itoh, N. , Seto, H. , & Dairi, T. (2008). An alternative menaquinone biosynthetic pathway operating in microorganisms. Science, 321, 1670–1673. 10.1126/science.1160446 18801996

[mmi14456-bib-0015] Kang, Y. , McMillan, I. , Norris, M. H. , & Hoang, T. T. (2015). Single prokaryotic cell isolation and total transcript amplification protocol for transcriptomic analysis. Nature Protocols, 10, 974–984. 10.1038/nprot.2015.058 26042386 PMC4494743

[mmi14456-bib-0016] Kopfmann, S. , Roesch, S. K. , & Hess, W. R. (2016). Type II toxin‐antitoxin systems in the unicellular *cyanobacterium Synechocystis* sp. PCC 6803. Toxins, 8, 228 10.3390/toxins8070228 PMC4963859 27455323

[mmi14456-bib-0017] Kroger, C. , Dillon, S. C. , Cameron, A. D. S. , Papenfort, K. , Sivasankaran, S. K. , Hokamp, K. , … Hinton, J. C. D. (2012). The transcriptional landscape and small RNAs of *Salmonella enterica* serovar Typhimurium. Proceedings of the National Academy of Sciences of the United States of America, 109, E1277–E1286. 10.1073/pnas.1201061109 22538806 PMC3356629

[mmi14456-bib-0018] Lasa, I. , Toledo‐Arana, A. , Dobin, A. , Villanueva, M. , de los Mozos, I. R. , Vergara‐Irigaray, M. , … Gingeras, T. R. (2011). Genome‐wide antisense transcription drives mRNA processing in bacteria. Proceedings of the National Academy of Sciences of the United States of America, 108, 20172–20177. 10.1073/pnas.1113521108 22123973 PMC3250193

[mmi14456-bib-0019] Lasa, I. , Toledo‐Arana, A. , & Gingeras, T. R. (2012). An effort to make sense of antisense transcription in bacteria. RNA Biology, 9, 1039–1044. 10.4161/rna.21167 22858676 PMC3551857

[mmi14456-bib-0020] Lasa, I. , & Villanueva, M. (2014). Overlapping transcription and bacterial RNA removal. Proceedings of the National Academy of Sciences of the United States of America, 111, 2868–2869. 10.1073/pnas.1324236111 24550470 PMC3939905

[mmi14456-bib-0021] Lioliou, E. , Sharma, C. M. , Caldelari, I. , Helfer, A.‐C. , Fechter, P. , Vandenesch, F. , Vogel, J. , & Romby, P. (2012). Global regulatory functions of the Staphylococcus aureus endoribonuclease III in gene expression. PLoS Genetics, 8(6), e1002782 10.1371/journal.pgen.1002782. 22761586 PMC3386247

[mmi14456-bib-0022] Lybecker, M. , Zimmermann, B. , Bilusic, I. , Tukhtubaeva, N. , & Schroeder, R. (2014). The double‐stranded transcriptome of *Escherichia coli* . Proceedings of the National Academy of Sciences of the United States of America, 111, 3134–3139. 10.1073/pnas.1315974111 24453212 PMC3939876

[mmi14456-bib-0023] Mellin, J. R. , Tiensuu, T. , Bécavin, C. , Gouin, E. , Johansson, J. , & Cossart, P. (2013). A riboswitch‐regulated antisense RNA in *Listeria monocytogenes* . Proceedings of the National Academy of Sciences of the United States of America, 110, 13132–13137. 10.1073/pnas.1304795110 23878253 PMC3740843

[mmi14456-bib-0024] Mitschke, J. , Georg, J. , Scholz, I. , Sharma, C. M. , Dienst, D. , Bantscheff, J. , … Hess, W. R. (2011). An experimentally anchored map of transcriptional start sites in the model cyanobacterium *Synechocystis* sp. PCC6803. Proceedings of the National Academy of Sciences of the United States of America, 108, 2124–2129. 21245330 10.1073/pnas.1015154108 PMC3033270

[mmi14456-bib-0025] Proctor, R. A. , Kriegeskorte, A. , Kahl, B. C. , Becker, K. , Löffler, B. , & Peters, G. (2014). *Staphylococcus aureus* Small Colony Variants (SCVs): A road map for the metabolic pathways involved in persistent infections. Frontiers in Cellular and Infection Microbiology, 4, 99 10.3389/fcimb.2014.00099 25120957 PMC4112797

[mmi14456-bib-0026] Quereda, J. J. , & Cossart, P. (2017). Regulating bacterial virulence with RNA. Annual Review of Microbiology, 71, 263–280. 10.1146/annurev-micro-030117-020335 28886688

[mmi14456-bib-0027] Quiles‐Puchalt, N. , Tormo‐Más, M. Á. , Campoy, S. , Toledo‐Arana, A. , Monedero, V. , Lasa, Í. , … Penadés, J. R. (2013). A super‐family of transcriptional activators regulates bacteriophage packaging and lysis in Gram‐positive bacteria. Nucleic Acids Research, 41, 7260–7275. 10.1093/nar/gkt508 23771138 PMC3753634

[mmi14456-bib-0028] Ruiz de los Mozos, I. , Vergara‐Irigaray, M. , Segura, V. , Villanueva, M. , Bitarte, N. , Saramago, M. , … Toledo‐Arana, A. (2013). Base pairing interaction between 5′‐ and 3′‐UTRs controls *icaR* mRNA translation in *Staphylococcus aureus* . PLoS Genetics, 9, e1004001 10.1371/journal.pgen.1004001 24367275 PMC3868564

[mmi14456-bib-0029] Sáenz‐Lahoya, S. , Bitarte, N. , García, B. , Burgui, S. , Vergara‐Irigaray, M. , Valle, J. , … Lasa, I. (2019). Noncontiguous operon is a genetic organization for coordinating bacterial gene expression. Proceedings of the National Academy of Sciences of the United States of America, 116, 1733–1738. 10.1073/pnas.1812746116 30635413 PMC6358700

[mmi14456-bib-0030] Saliba, A.‐E. , Santos, S. C. , & Vogel, J. (2017). New RNA‐seq approaches for the study of bacterial pathogens. Current Opinion in Microbiology, 35, 78–87. 10.1016/j.mib.2017.01.001 28214646

[mmi14456-bib-0031] Saliba, A.‐E. , Westermann, A. J. , Gorski, S. A. , & Vogel, J. (2014). Single‐cell RNA‐seq: Advances and future challenges. Nucleic Acids Research, 42, 8845–8860. 10.1093/nar/gku555 25053837 PMC4132710

[mmi14456-bib-0032] Serganov, A. , & Nudler, E. (2013). A decade of riboswitches. Cell, 152, 17–24. 10.1016/j.cell.2012.12.024 23332744 PMC4215550

[mmi14456-bib-0033] Sesto, N. , Wurtzel, O. , Archambaud, C. , Sorek, R. , & Cossart, P. (2013). The excludon: A new concept in bacterial antisense RNA‐mediated gene regulation. Nature Reviews Microbiology, 11, 75–82. 10.1038/nrmicro2934 23268228

[mmi14456-bib-0034] Sharma, C. M. , Hoffmann, S. , Darfeuille, F. , Reignier, J. , Findeiß, S. , Sittka, A. , … Vogel, J. (2010). The primary transcriptome of the major human pathogen *Helicobacter pylori* . Nature, 464, 250–255. 10.1038/nature08756 20164839

[mmi14456-bib-0035] Taboada, B. , Estrada, K. , Ciria, R. , & Merino, E. (2018). Operon‐mapper: A web server for precise operon identification in bacterial and archaeal genomes. Bioinformatics, 34, 4118–4120. 10.1093/bioinformatics/bty496 29931111 PMC6247939

[mmi14456-bib-0036] Thomason, M. K. , & Storz, G. (2010). Bacterial antisense RNAs: How many are there, and what are they doing? Annual Review of Genetics, 44, 167–188. 10.1146/annurev-genet-102209-163523 PMC3030471 20707673

[mmi14456-bib-0037] Toledo‐Arana, A. , Dussurget, O. , Nikitas, G. , Sesto, N. , Guet‐Revillet, H. , Balestrino, D. , … Cossart, P. (2009). The *Listeria* transcriptional landscape from saprophytism to virulence. Nature, 459, 950–956. 10.1038/nature08080 19448609

[mmi14456-bib-0038] von Eiff, C. , Heilmann, C. , Proctor, R. A. , Woltz, C. , Peters, G. , & Götz, F. (1997). A site‐directed *Staphylococcus aureus hemB* mutant is a small‐colony variant which persists intracellularly. Journal of Bacteriology, 179, 4706–4712. 10.1128/JB.179.15.4706-4712.1997 9244256 PMC179315

[mmi14456-bib-0039] Wade, J. T. , & Grainger, D. C. (2014). Pervasive transcription: Illuminating the dark matter of bacterial transcriptomes. Nature Reviews Microbiology, 12, 647–653. 10.1038/nrmicro3316 25069631

[mmi14456-bib-0040] Winkler, W. C. , & Breaker, R. R. (2005). Regulation of bacterial gene expression by riboswitches. Annual Review of Microbiology, 59, 487–517. 10.1146/annurev.micro.59.030804.121336 16153177

[mmi14456-bib-0041] Wurtzel, O. , Sesto, N. , Mellin, J. R. , Karunker, I. , Edelheit, S. , Bécavin, C. , … Sorek, R. (2012). Comparative transcriptomics of pathogenic and non‐pathogenic *Listeria* species. Molecular Systems Biology, 8, 583 10.1038/msb.2012.11 22617957 PMC3377988

[mmi14456-bib-0042] Yan, B. , Boitano, M. , Clark, T. A. , & Ettwiller, L. (2018). SMRT‐Cappable‐seq reveals complex operon variants in bacteria. Nature Communications, 9, 3676 10.1038/s41467-018-05997-6 PMC6131387 30201986

